# Soil moisture-based irrigation interval and irrigation performance evaluation: In the case of lower kulfo catchment, Ethiopia

**DOI:** 10.1016/j.heliyon.2024.e36089

**Published:** 2024-08-10

**Authors:** Birara Gebeyhu, Samuel Dagalo, Mekuanent Muluneh

**Affiliations:** a Arba Minch Water Technology Institute, Faculty of Water Resources and Irrigation Engineering, Arba Minch University, Arba Minch, Ethiopia; b Arba Minch Water Technology Institute, Water Resources Research Centre, Arba Minch University, Arba Minch, Ethiopia

**Keywords:** Crop productivity, Irrigation adequacy, Irrigation equity, Irrigation interval, Moisture depletion

## Abstract

The lack of soil moisture-based irrigation intervals, poor distribution of irrigation water among users, and the time-based and spatial variability of water supply have been challenges for the productivity of irrigation schemes in the Lower Kulfo catchment, Southern Ethiopia. This study was conducted to develop soil moisture-based irrigation intervals and to evaluate irrigation water delivery and field level irrigation efficiencies. Soil water content, and flow along the canal and in the field were measured directly, and irrigation duty was estimated by using CropWat 8 model. To minimize water stress or excess problems, irrigation needs to be applied when soil water content drops to 35.7 % for onion and pepper, 34.4 %% for watermelon, and 32.5 % for wheat and maize from field capacity. However, irrigation was applied at 36.2 % for onion, 35.4 % for watermelon, 36.4 % for pepper, 36.2 % for maize, and 35 % for wheat in the existing irrigation scheme that increased irrigation amount in the field. The average percentage of soil moisture depletion (p) at time of irrigation was 27.4 %, which was below the recommended value. The average adopted irrigation and design irrigation interval were 4 & 6 days for onion and pepper, 5 & 7 days for watermelon and wheat, and 6 & 7 days for maize, respectively. The mean irrigation adequacy and dependability of the irrigation scheme in the lower Kulfo catchment were 1 & 0 for Arba Minch irrigation scheme, 0.5 & 0.2 for Arba Minch University farmland, 0.4 & 0.25 for private farmland and 0.1 & 0.43 for Kollashara farmland, respectively. The value of irrigation equity was 0.7 in January, 0.6 in February, and 0.8 in March which indicates the highly temporary variation of irrigation adequacy. The mean value of canal conveyance was 82.7 % and the average on-farm irrigation efficiency also was 56.6 %. The average value crop yield in the present study were 0.5ton/ha for wheat, 4.9ton/ha for onion, 6.2ton/ha for pepper, 0.6ton/ha for watermelon, 4.2ton/ha for maize that was very low compared with other control irrigation in the study area. Inadequate soil moisture-based intervals, inequitable water distribution, and variable supply hinder irrigation in the Lower Kulfo catchment; adopting optimized practices and robust management can enhance efficiency, equity, and crop productivity.

## 1 Introduction

Irrigation plays a pivotal role in Ethiopia's economic development by supporting agricultural growth, rural livelihoods, and export revenues while enhancing resilience to climate change impacts [1]. About 1.5 million hectares of land available for irrigation in Ethiopia, covering large and small-scale irrigation scheme across the various regions [2]. Modern irrigation in Ethiopia began in the early 20th century, gaining momentum in the 1950s and 1960s with key projects in the Awash and Blue Nile Basins, Koka Dam, and Rift Valley [3]. Supported by government and international aid, these initiatives aimed to enhance agricultural productivity and economic growth, laying the foundation for ongoing advancements to meet growing food and economic needs [4].

Soil moisture monitoring before and after irrigation plays a important role in optimizing irrigation practices, conserving water, improving profit of crop [5], and preserving soil health [6]. It enables farmers to optimize irrigation timing, and water application, resulting in efficient resource use and improved agricultural results [7]. The percentage of soil moisture depletion evaluation in irrigation applications helps optimize water use, improve crop yield, and enhance overall agricultural sustainability [8]. Soil moisture-based irrigation scheduling is a method that uses real-time soil moisture data to determine when and how much to irrigate [9]. Field capacity and permanent wilting points are important for efficient irrigation scheduling, avoiding water stress, optimizing use, and enhancing crop yield [10].

Irrigation water distribution indicators measure the system's ability to supply water at the necessary rate at the appropriate location and time. The key indicators of water delivery performance are adequacy, equity, dependability, and conveyance efficiencies [11]. Based on [12], Adequacy refers to the relationship between water supply to the crop and its demand and when supply precisely meets demand, adequacy is considered as one. Irrigation adequacy may decrease with deficit irrigation, either by delivering less water than the crop's maximum water requirement or by extending the interval between irrigation cycles [13]. Equity in water supply refers to how each person's share appears or is viewed to be equitable by all system users [14]. If every site has a comparable quantity of water or an adequate supply, there will be a perfectly equitable distribution [15]. Equity refers to the extent of variation in relative water distribution across different points within the irrigated area [15].

Dependability in irrigation water systems, defined by the consistency of water delivery relative to scheduled amounts, is vital for effective irrigation management [16]. According to Ref. [17], a canal system that consistently delivers slightly less water may be more beneficial than one with unpredictable but average delivery. The variability in water delivery requirements across different regions characterizes the dependability of water delivery [15]. Assessing the efficiency of irrigation canals is essential for sustainable water management and effective agricultural development, as it helps in reducing water wastage [18]. Key parameters such as application efficiency, storage efficiency, and percolation are vital for measuring the effectiveness of irrigation water application and the quantity of water stored in the crop root zone [19].

Temporary and spatial variability of irrigation adequacy leads to water scarcity, conflicts among users, and reduced agricultural productivity [20]. Ensuring fairness in water distribution remains a significant challenge within intermittent water supply systems of irrigation schemes [21]. Water for irrigation is not always distributed and allocated fairly across users [22]. Climate change significantly impacts irrigation adequacy through altered precipitation patterns, increased evapotranspiration, and variable water availability from melting glaciers and changing river flows [23].

Irrigated agriculture plays a essential role in the Kulfo Lower Catchment, Southern Ethiopia, sustaining livelihoods and ensuring food security amidst climatic challenges [24]. However, the irrigation practise in the region was subject with different challenges such as lack of proper irrigation intervals, inefficient water allocation among the user, and adaptation of traditional methods of water application techniques. Additionally, water distribution is inequitable and highly variable among months due to climatic fluctuations. Downstream irrigation users like Kollashara Kebele small-hold farmers in the lower Kulfo catchment used untreated wastewater for irrigation that flowed from Arba Minch University due lack of irrigation water from the sources. Adopted irrigation intervals in the lower Kulfo catchment are not decided based on proper irrigation scheduling. This indicates that the scheme needs soil moisture-based irrigation interval based on soil field capacity and actual soil water at the time of irrigation. Despite these critical challenges, no comprehensive research has been conducted to evaluate these problems and suggest mitigation measures. This research is motivated by the urgent need to address these issues to enhance agricultural productivity and support local livelihoods. Therefore, this research was conducted to develop soil moisture-based irrigation intervals and to evaluate performance indicators of irrigation water allocation and application that promote equitable water distribution, enhance accessibility, and provide data-driven recommendations for policy and management improvements.

## 2 Material and METHODOLOGY

### 2.1 Description of the study area

**Fig. 1:**
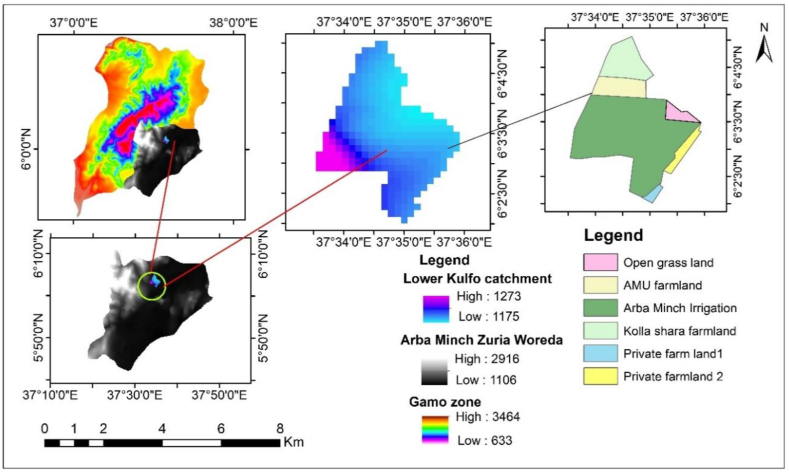
Study area location.

The Lower Kulfo catchment is situated between latitudes 6° 2′ 30″ and 6° 4′ 30″ N, and longitudes 37° 33′ 0″ and 37° 36′ 0″ E in southern Ethiopia (Fig. 1). This area, ranging in elevation from 1175 to 1273 m above sea level, is located 455 km from Addis Ababa. The catchment encompasses the Arba Minch irrigation scheme, Arba Minch University farmland, smallholder farms in the Kolla Shara district, and private farms near Arba Minch airport. The irrigated areas include 835.22 ha for the Arba Minch irrigation scheme, 109.17 ha for the university farm, 160.23 ha for Kollashara farmland, and 18.44 ha and 52.76 ha for two private farmlands, totaling 1175.82 ha. Major crops grown in this area are wheat, maize, pepper, onion, watermelon, and banana.

**Fig. 2:**
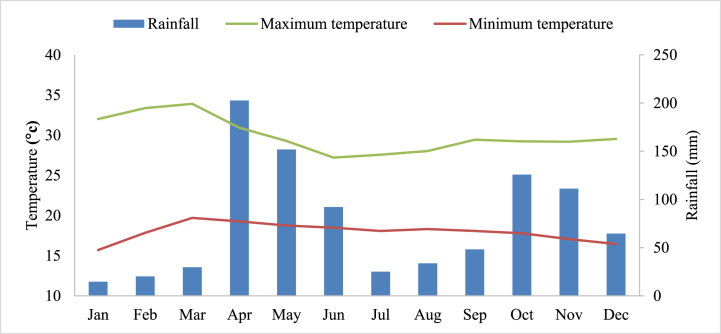
Average monthly minimum temperature, maximum temperature, and rainfall.

The average temperature in the study area was 23.3 °C (Fig. 2) and the mean monthly reference evapotranspiration was 4.25 mm/day, with the highest values occurring in April and the lowest in December.

The rainfall distribution season of the study area was bimodal and these rainy seasons were from April to Jun and October to December and the total received average annual rainfall was 904 mm. The common rainy season in Ethiopia was from June to September and the climate condition of the Arba Minch area was different from this agroclimatic condition. The irrigation season of the present study was from January to the end of March which was the driest season in the Arba Minch area.

### 2.2 Data collection and analysis

#### 2.2.1 Soil physiochemical properties

The fixed dimensions of the area sampling plots were 10 × 90m for onions, 20 × 50m for maize, and 30 × 40m for wheat. Watermelon and pepper had average furrow areas of 15.8 m^2^ and 3.7 m^2^, respectively, with furrow lengths of 20 m and 6 m. Composite soil samples were taken to a depth of 90 cm using an auger, with intervals of 30 cm. Soil texture and bulk density were assessed via hydrometer and oven-dry methods. Field capacity and permanent wilting point were measured with a pressure plate apparatus. Soil infiltration rates were gauged with a double ring infiltrometer, and soil water content before and after irrigation was estimated using time-domain reflectometer (TDR), with post-irrigation measurements taken 24–72 h later. Those estimated values soil water used to calculate irrigation required and soil water storage after irrigation [25]. The depth of irrigation needed (Zr) is calculated as the difference between field capacity and the current soil moisture in the root zone before irrigation [26], as shown in Eq (1). This measurement is then used to determine the appropriate irrigation interval.

(1) $$Zr=\sum_{i=1}^{n}\left(\frac{\Theta fc-\Theta i}{100}\right)*D_{i}\;$$

Where; Zr = depth of irrigation required (mm), өf_c_ = soil field capacity (%), ө_i_ = initial volumetric soil water before the irrigation (%), D_i_ = ith layer of crop root depth (mm) and n = number of soil layers.

The quantity of water that remains in the crop's root zone following irrigation is known as soil water storage, and it was calculated using [27] as shown in Eq (2).

(2) $$Ds=\sum_{i=1}^{n}\left(\frac{\theta_{ai}-\theta_{bi}\Theta }{100}\right)*{RD}_{i}\;$$

Where; θ_ai_ and θ_bi_ are the moisture content of the ith soil layer after and before irrigation (%), respectively.

In order to calculate the soil water content before and after irrigation, as shown in Eq. (3), the root depth of the crops (RDt) at the time of irrigation was assessed using [28].

(3) $${RD}_{t}={RD}_{m}\left[(0.5+0.5*\sin\left(3.03*tr-1.47\right)\right]$$

Where: tr, RD_m_, RD_t_, and tm represent the relative time (t/tm), the maximum predicted rooting depth of crops (cm), the rooting depth on day t (cm), and the time to physiological maturity (days after sowing), respectively.

The percentage of soil moisture depletion from field capacity (p) was estimated by the following Eq (4) and that was compared with FAO 56 recommended value and this value was used to understand when irrigation will be applied.

(4) $$p\;\left(\%\right)=\left(\frac{FC-\theta t}{FC-PWP}\right)*100$$

Where FC, θt, and PWP represent value field capacity (% vol), soil water at the time of irrigation (% vol), and permanent wilting point (% vol), respectively.

#### 2.2.2 Reference and crop evapotranspiration, and effective rainfall

Based on [29], potential or reference evapotranspiration (ET_o_), crop water requirement (ET_c_), and effective rainfalls (P_e_) were evaluated by using Eq (5), Eq (6), and Eq (7)or Eq (8) from climate data through the CropWat 8.0 model. Additionally, the maximum irrigation duty for the actual crop pattern was assessed using the CropWat model, which also determined the required discharge at the canal inlet (QR).

(5) $${ET}_{o}=\frac{0.408\Delta \left(Rn+G\right)+r*\frac{900}{T+273}u2\left(es-ea\right)}{\Delta +r\left(1+0.34u2\right)}$$

Where; U_2_ is the wind speed at a height of 2 m (ms-1), e_s_ is thesaturation vapour pressure (kPa), e_a_ is the actual vapour pressure (kPa), (e_s_ -e_a_) is the saturated vapour pressure deficit (kPa), Δ is the slope vapour pressure curve (kPa oc-1), and r is the psychrometric constant (kPa oc-1).

(6) $${ET}_{c}=\text{kc}*\;{ET}_{o}$$

Where; Kc is crop coefficients that were collected from irrigation and drainage paper (FAO 56)

(7) $$P_{e}=\frac{\left(p*\left(125-0.2*3*p\right)\right)}{125}$$

ifp≤250/3

(8) $$P_{e}=\frac{250}{3}+0.1p$$

;ifp>250/3

Irrigation requirement (IR) was estimated by subtracting effective rainfall from crop water requirement as presented in Eq (9).

(9) $$\text{IR}={ET}_{c}-P_{e}$$

The estimated irrigation requirement (IR) was used as input to estimate irrigation duty through CropWat model and the duty was used to estimate the required discharge of the canal inlet.

#### 2.2.3 Flow measurement

Delivered discharge to farm inlet was measured by using flume and the depth of applied water to the field (Da) was estimated by using Eq (10).

(10) $$\text{Da}\;\left(\text{mm}\right)=\frac{Qt}{W*L}$$

Where, t is duration of irrigation (sce), W is width of furrow or border (m), and L is length furrow or border (m)

Velocity canal flow was measured by using current meter at the upper, mid, and lower reach of canal and delivered discharge at the canal inlet (QDA) was estimated through Eq (11).

(11) $$QDA=\sum_{n=1}^{\infty }\left(b_{1}\left(\frac{v_{1}+v_{2}}{2}\right)*\frac{d_{1}+d_{2}}{2}\ldots .+b_{n}\left(\frac{v_{n-1}+v_{n}}{2}\right)*\frac{d_{n-1}+d_{n}}{2}\right)\;$$

Where; QD is delivered discharge (m^3^/s), b is canal width at n-segment (m), V is canal velocity at n-segment of canal cross-section (m/s), d is depth at n-segment of canal cross-section (m).

### 2.3 Irrigation interval

Soil moisture-based irrigation intervals contribute to more precise and sustainable agricultural practices, benefiting both farmers and the environment [30] and that was estimated by Eq (12).

(12) $$\text{Intended}\;\text{Irrigation}\;\text{interval}\left(\text{days}\right)=\frac{Zr}{{ET}_{c}}$$

Where Zr is depth of irrigation required as estimated in Eq (1) and ET_c_ was the daily maximum crop evapotranspiration (mm/day) that was estimated through the CropWat model.

### 2.4 Irrigation performance

#### 2.4.1 Water allocation performance

A. Adequacy (PA)

The adequacy index displays the degree to which the total water deliveries is enough to satisfy crop water requirement in a specific growing season. During the crop growing period, adequacy was determined for head, middle, and tail reach of the irrigation schemes in the lower kulfo catchment over the time consideration (T), and adequacy indicator (PA) was determined based on [31] as expressed by Eq (13).

(13) $$P_{A}=\frac{1}{T}\;\sum_{T}\left(\frac{1}{R}\sum P_{A\;}\right),\left\{ \begin{array}{c} P_{A}=\frac{QDA}{QRP},if\;QDA\leq QRP\\ P_{A}=1,if\;QDA>QRP \end{array}\right.\;$$

Where; QD is the actual amount of water provided by the system, QR is the amount of water needed for crop consumptive use, T is time, R is the location of canals.

The required discharge at the canal inlet (QRP) for each irrigation user in the lower kulfo catchment was estimated based on [32] as described in Eq (14) and the duty of irrigation for the dominant crop was evaluated using CropWat model and time factors were also estimated by using Eq (15).

(14) $$\text{Required}\;\text{Discharge}\;\text{at}\;\text{canal}\;\text{inlet}\;\left(\text{QRP}\right)=\frac{A*D*T}{IE}$$

(15) $$\text{Time}\;\text{factor}\;\left(T\right)=\frac{Total\;available\;hour\;}{hours\;of\;irrigation}$$

Where; A = irrigated area covered by irrigated canal (ha), D = irrigation duty (l/s/ha), T = Time factors (fraction) and IE = irrigation efficiency (fraction).

B. Dependability (PD)

According to Ref. [33], dependability is the degree of the spatial variability of the irrigation delivery in comparison with the requirements and that was estimated based on [34] as presented in Eq (16) and coefficient of temporal variation was estimated by using Eq 17

(16) $$\text{PD}=\frac{1}{R}\sum_{R=1}^{R}\left(\text{CVT}\left(\text{PA}\right)\right)$$

(17) $$\text{CVT}=\frac{\text{SD}}{\text{mean}}$$

Where, SD= Standard deviation and CVT(QDA/QRP) is coefficient of temporal variation of the ratio in period T.

When the value of dependability approaches to zero, it indicates that the water delivery is working properly for the time [35], and the reverse is true when the value of PD becomes above 0.2 which means the water delivery is not working properly.

C. Equity (PE)

Equity of water distribution is a share of each individual or considered fair by all the system members [21] and it is the temporal variation of irrigation over the location of the canal region [36] and it was estimated by Eq (18).

(18) $$PE=\frac{1}{T}\sum_{T}CVR\left(\frac{QDA}{QRP}\right)$$

Where, CVR is the spatial coefficient of variation of the ratio QDA to QRP over the region R. Closer the value of PE is to zero, the greater the degree of equity in delivery. Performance is said to be good when the value of PE is between 0.00 and 0.10, fair when it is between 0.11 and 0.25, and poor when it is more than 0.25 [31].

D. Conveyance efficiency

Canal conveyance efficiency (Ec) is defined as the ratio of the amount of water that reaches the field inlet to the total amount of water diverted from the sources into the irrigation system [37]. It was used to evaluate the efficiency of the canal system in conveying irrigation water [38] and conveyance efficiencies were estimated according to Ref. [37] through Eq (19).

(19) $$\text{Conveyance}\;\text{efficiency}\;\left(\text{Ec}\right)=100*\frac{Discharge\;at\;outlet}{Discharge\;at\;the\;inlet}$$

#### 2.4.2 On-farm performance efficiencies

Application and storage efficiency are the essential parameters to measure the effectiveness of irrigation water application and the quantity of water stored in the crop root zone [39]. Field level irrigation efficiencies such as application, storage, percolation loss, and overall efficiencies were evaluated based on [27,39,40] by using Eq (20), Eq (21), Eq (22), and Eq (23), respectively.

(20) $$\text{Application}\;\text{efficiency}\;\left(\text{Ea}\right)=100*\frac{Ds}{Da}$$

(21) $$\text{Storage}\;\text{efficiency}\;\left(E_{s}\right)=100*\frac{\text{Ds}}{\text{Zr}}$$

(22) $$\text{Percolation}\;\text{loss}\left(P\right)=100*\frac{Da-Ds\;}{Da}$$

(23) $$\text{Overall}\;\text{irrigation}\;\text{efficiency}\;\left(\text{Eo}\right)=\frac{Ea*EC}{100}$$

Where: Ds is depth soil water storage depth (mm), Da is applied irrigation depth (mm), and Zr depth of irrigation required (mm).

### 2.5 Crop productivity

Water and land productivity are vital elements in longer-term strategic water resources planning [41] and that was estimated through Eqs (24), (25)).

(24) $$\text{Water}\;\text{productivity}\;\left(\text{WP}\right)=\frac{\text{Yield}\;\left(\text{kg}\right)}{\text{Total}\;\text{applied}\;\text{water}\;\left(m^{3}\right)}$$

(25) $$\text{Land}\;\text{Productivity}\;\left(\text{LP}\right)=\frac{\text{Yield}\;\left(\text{ton}\right)}{\text{Irrigated}\;\text{land}\;\left(\text{ha}\right)}$$

## 3 Result and discussion

### 3.1 Soil phyiscal propeties

Textural class of soil type in the present study was clays with 1.29 gm/cm^3^ dry bulk density (Table 1). The bulk density of soil in the present study was shown increasing trends with soil depth that may be due to compaction and natural processes. In the current study, the average field capacity and permanent wilting point were 39.5 and 26.8 %, respectively, while the average total available water also was 12.7 %. Volumetric permanent wilting point and field capacity of clay soil were 25 % & 40 % [42], 21.82 % & 35.03 % [43], and 21.2 % & 44.8 % [44], respectively. The value of the current study differed slightly from that of an earlier study, possibly as a result of the irrigated farms' field management status.

**Table 1:** Soil physical properties.

Users	Depth (cm)	Texture	BD (gm/cm3)	FC (%vol)	PWP (%vol)
	0–30	Clay	1.25	39	27.0
Arba Minch irrigation scheme	30–60	Clay	1.31	38.8	26.4
	60–90	Clay	1.36	38.3	25.2
	0–30	Clay	1.21	40.7	28
Arba Minch University farmland	30–60	clay	1.28	40.4	28.1
	60–90	Clay	1.35	40.2	28.2
	0–30	silty clay	1.24	40.2	25.6
Kolla shara farmland	30–60	Clay	1.22	36.5	24.1
	60–90	Clay	1.34	39.3	27
	0–30	Clay	1.27	40.3	26.8
Private farmland	30–60	Clay	1.31	40.0	27.5
	60–90	Clay	1.37	39.9	27.6
	**Average**	**Clay**	**1.29**	**39.5**	**26.8**

Where; BD is bulk density, Fc is field capacity and PWP is permeant wilting point.

**Fig. 3:**
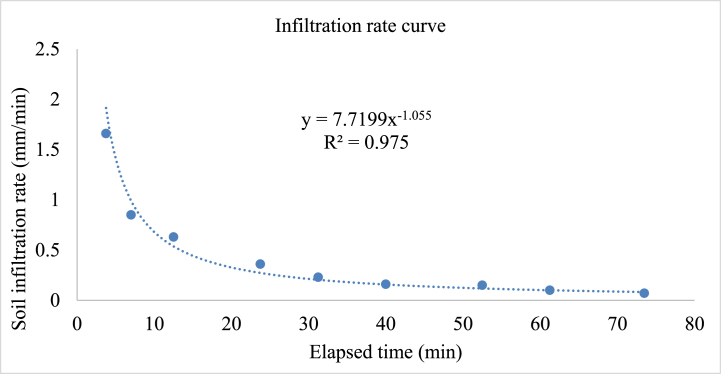
Soil infiltration rate.

The average maximum and basic infiltration rate of soil in the present study was 1.7 and 0.1 mm/min or 75.6 and 3.6 mm/h, respectively (Fig. 3). This reported soil infiltration rate of current was lies between the basic infiltration rates of 2–5 mm/h for clay soil recommended [45]. This estimated soil basic soil infiltration is important for managing irrigation application rate in lower Kulfo catchment because it helps in determining how quickly water can infiltrate into the soil [46]. Therefore, irrigation application rate in lower kulfo catchment should be less than or equal to soil basic infiltration rate to minimize runoff problem in the scheme period.

### 3.2 Soil water depletion

**Fig. 4:**
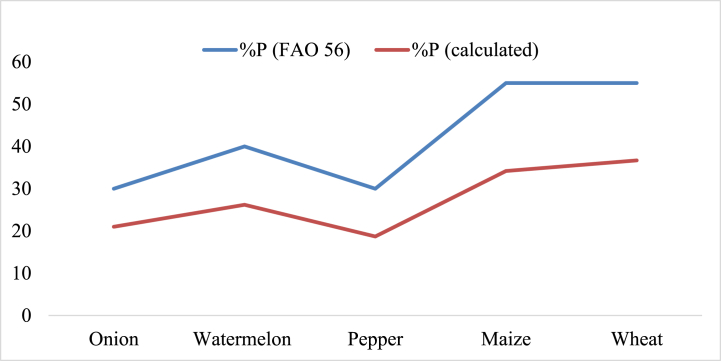
percentage of soil moisture depletion (%P) and recommended value (%P from FAO) of major crop in lower Kulfo catchment.

Calculated soil moisture depletion for onion, watermelon, pepper, maize, and wheat is consistently lower than FAO 56 recommendations, implying more frequent irrigation (Fig. 4). The average percentage of soil moisture depletion (P) was 27.4 % lower than the recommended value, leading to higher waterlogging problem. To avoid water stress and excess problems, irrigation can apply when the soil water content drops to 35.7 % for onion and pepper, 34.4 % for watermelon, and 32.5 % for maize and wheat from field capacity. However, irrigation was applied at different soil water contents: 36.2 % for onion, 35.4 % for watermelon, 36.4 % for pepper, 36.2 % for maize, and 35 % for wheat in existing irrigation practices that were deviating from the recommended levels. This finding highlights the importance of precise irrigation management to optimize water use efficiency and mitigate the risk of waterlogging. Adjust irrigation timing to align with soil moisture levels recommended by FAO 56, ensuring precise irrigation management and optimizing water use efficiency.

### 3.3 Soil water content

#### 3.3.1 Onion

**Fig. 5:**
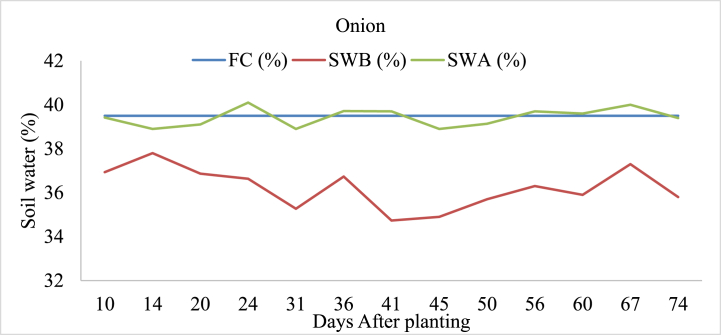
Soil field capacity (FC), soil water content before irrigation (SWB), and soil water content after irrigation (SWA) in onion-irrigated land.

A detailed analysis shows that waterlogging occurred in onion-irrigated land on day 24 after the planting, where the soil water after irrigation (SWA) was recorded at 40.1 % exceeding from field capacity of the soil (Fig. 5). This indicates that the soil retained more water than its field capacity, leading to waterlogged conditions. Subsequent days with potential waterlogging include day 56 and day 67, with SWA values of 39.7 % and 40.0 %, respectively. These instances suggest periodic excess water retention in the soil, which could impact onion crop growth and development.

#### 3.3.2 Watermelon

**Fig. 6:**
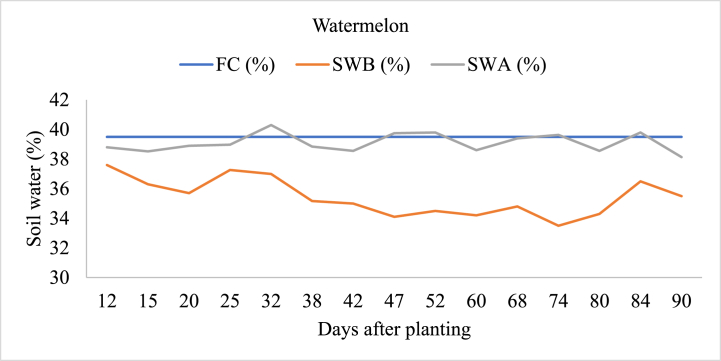
Soil field capacity (FC), soil water content before irrigation (SWB), and soil water content after irrigation (SWA) in watermelon-irrigated land.

As presented in Fig. 6, fluctuations in soil water content over time were observed in watermelon irrigated. Initially, soil water before irrigation (SWB) ranged from 37.6 % to 33.5 % across different days after planting (DAP). Following irrigation, soil water after irrigation (SWA) varied from 36.6 % to 40.3 %. Notably, on day 32 (DAP = 32), we observed a peak in SWA at 40.3 %, suggesting a potential risk of waterlogging due to excessive moisture accumulation. The mean soil water after irrigation under control full irrigation level was 39.4 % [47] which approached to soil field capacity.

#### 3.3.3 Pepper

**Fig. 7:**
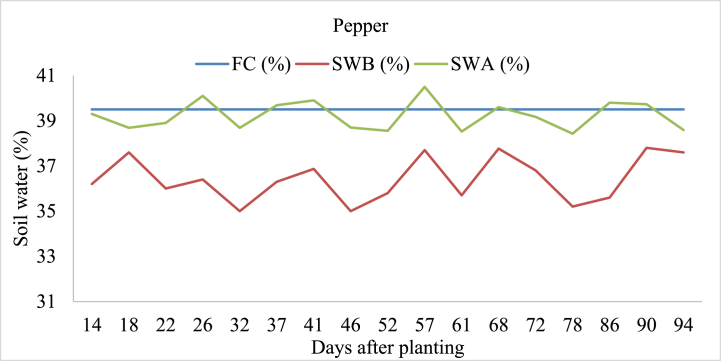
Soil field capacity (FC), soil water content before irrigation (SWB), and soil water content after irrigation (SWA) in pepper-irrigated land.

Soil water in pepper irrigated before irrigation (SWB) ranged from 35.0 % to 37.8 %, reflecting variations in water availability at the start of each irrigation cycle (Fig. 7). Following irrigation events, SWA % values showed fluctuations between 38.4 % and 40.5 %, and soil water after irrigation was above field capacity at 26, 37, 41, 57, 68, 86, and 90 days after the planting. These observations highlight the importance of precise irrigation management in optimizing water use efficiency and supporting healthy pepper crop development. Exceeding soil water storage above field capacity in irrigated land leads to waterlogging, nutrient leaching, reduced root growth, increased erosion, and degraded soil structure, harming plant health and productivity [48].

#### 3.3.4 Maize

**Fig. 8:**
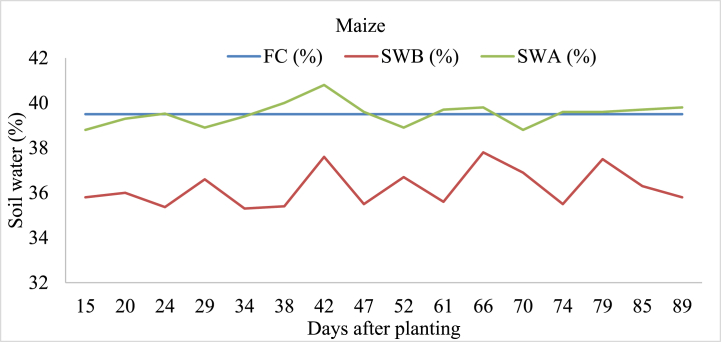
Soil field capacity (FC), soil water content before irrigation (SWB), and soil water content after irrigation (SWA) in maize-irrigated land.

Soil moisture content before irrigation (SWB) in maize-irrigated land varied slightly between 35.3 % and 37.8 %, while soil moisture levels after irrigation (SWA) fluctuated between 37.9 % and 40.8 % (Fig. 8). Similarly, soil water storage after irrigation on maize irrigated land was above the soil field capacity especially 42, 61, 66, 74, 74,79, and 89 days after the planting. These findings highlight that the adapted irrigation water management approach in the study area was poor. Over-irrigation causes drainage, waterlogging, nutrient leaching, reduced soil aeration, and impacting plant growth [49].

#### 3.3.5 Wheat

**Fig. 9:**
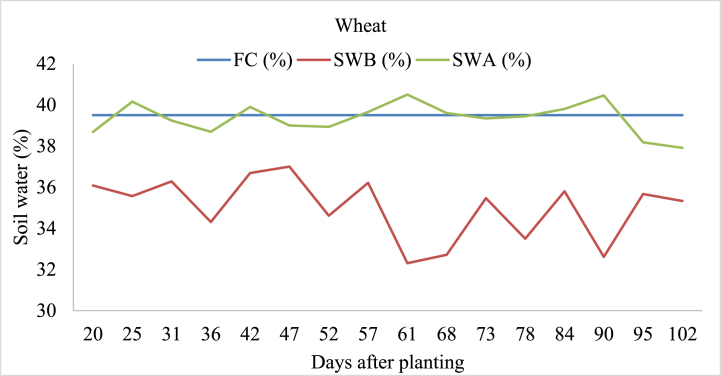
Soil field capacity (FC), soil water content before irrigation (SWB), and soil water content after irrigation (SWA) in wheat-irrigated land.

The study examined soil water dynamics in wheat fields under varying irrigation practices and natural precipitation. Before irrigation, soil water content (SWB) ranged from 32.3 % to 37.0 %, influenced by local irrigation techniques. Following irrigation, soil water content (SWA) increased to between 36.18 % and 40.50 %. Notably, SWA consistently exceeded the soil's field capacity at 25, 42, 61, and 90 days after planting, indicating crop aeration problem (Fig. 9). This highlights the significance of precise irrigation management tailored to local conditions to ensure adequate soil moisture, thereby maximizing crop yield and resource efficiency in agriculture.

### 3.4 Irrigation interval

The irrigation intervals currently adopted in the existing scheme were shorter than the soil moisture-based intervals estimated in the present study (Table 2). This discrepancy highlights the frequent application of irrigation that leads to excessive water loss through runoff and percolation, suggesting a need for optimizing irrigation schedules to enhance water use efficiency. The average adopted or user-based irrigation interval of the main crops in the area of Lower Kulfo was 5 days, while the intended interval was 7 days (Table 2). This indicates that irrigation was applied without considering the management allowable depletion point in the existing irrigation scheme practices. The average irrigation interval varied from 7 to 8 days (watermelon) [50], 5–7 days (pepper) [51], 7–10 days (onion) [52], 7–14 days (wheat) [53], and 7–10 days (maize) [54]. Therefore, the estimated soil moisture-based irrigation interval in the current study for each crop was found within ranges of earlier study.

**Table 2:** Adopted and intended irrigation interval of major crops in the lower kulfo catchment.

Crop type	Adopted interval	Intended interval
Onion	4	6
Watermelon	5	7
Wheat	5	7
Pepper	4	6
Maize	6	7
**Mean**	**5**	**7**

### 3.5 Actual delivered and required discharge

In January, the relatively low irrigation duty of 0.16 l/s/ha implied that crops require less water, possibly due to cooler temperatures or early growth stages that demand less moisture. In February it increased to 0.32 l/s/ha indicating a heightened need for water, which could be attributed to warmer weather, increased crop growth, or the beginning of critical growth stages. By March, the irrigation duty peaks at 0.41 l/s/ha, likely due to the continued rise in temperature as discussed in Fig. 2 and the crops reaching their most water-intensive growth phases. The time factor and irrigation efficiency used to calculate the required discharge in Eq (13) were 2 and 70 %, respectively. The comparison of actual delivery discharge (QDA) to peak required discharge (QRP) for different users in the Lower Kulfo catchment reveals significant disparities. For the Arba Minch irrigation scheme, QDA consistently exceeds QRP across all months (Table 3), indicating an over-delivery of water which could suggest inefficiencies or misallocation of resources. In contrast, the Arba Minch University (AMU), Kolla Shara, and private farms show a trend of under-delivery, where QDA frequently falls short of QRP. This under-delivery is particularly pronounced for Kolla Shara, with QDA being significantly lower than QRP, pointing to potential issues in water supply infrastructure or distribution strategies. Effective strategies could include infrastructure improvements, better scheduling of water deliveries, and adaptive management practices based on continuous monitoring of water demands and supply capabilities. The average required and delivered discharge in the Lower Kulfo catchment were 134.4 & 183.1 l/s (January), 268.8 & 450.4 l/s (February), and 344.3 & 537.4 l/s (March), respectively and the highest delivery of irrigation water occurred in March (Table 3).

**Table 3:** Actual delivered discharge (QDA) and required discharge (QRP).

Month	Jan		Feb		Mar	
Users/discharge	QRP (l/s)	QDA (l/s)	QRP (l/s)	QDA (l/s)	QRP (l/s)	QDA(l/s)
A/irrigation scheme	381.8	687.3	763.6	1680.0	978.4	2054.6
AMU farm land	49.9	25.0	99.8	59.9	127.9	51.2
Kolla shara	73.2	7.3	146.5	29.3	187.7	18.8
Private farm	32.5	13.0	65.1	32.5	83.4	25.0
**Mean**	**134.4**	**183.1**	**268.8**	**450.4**	**344.3**	**537.4**

### 3.6 Water allocation performance

#### 3.6.1 Adequacy (PA)

**Fig. 10:**
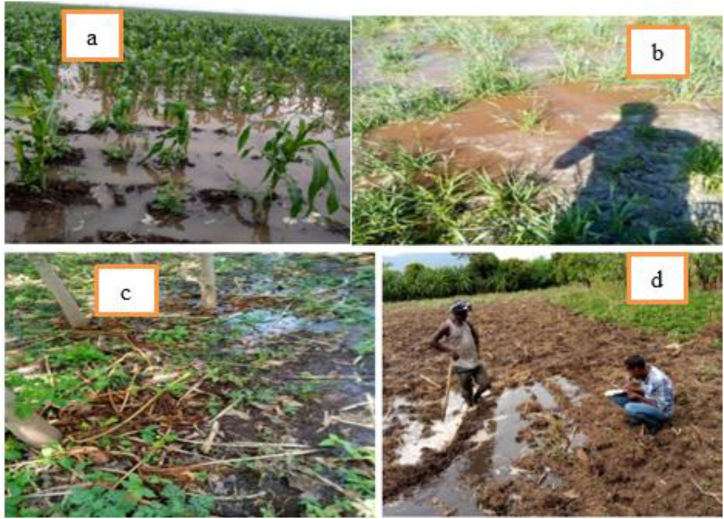
Excess irrigation on maize crop (a) and wheat crop (b) in Arba minch irrigation scheme, and wastewater irrigation in Arba Minch farmland (c) and Kolla shara farmland (d).

In January, the irrigation amounts are highest for the Arba Minch irrigation scheme, which indicates that this user group has adequate water supply for irrigation, likely meeting or exceeding their needs. All values of irrigation adequacy in the Arba Minch irrigation scheme were greater than one that shows excess irrigation loss. The average value irrigation adequacy was 1 (Arba Minch irrigation scheme), 0.5 (Farmland of Arba Minch University), 0.4 (farmland of private), and 0.2 (Kollashara kebele) as presented in Table 4. But another irrigation scheme in the lower Kulfo catchment especially Kollashara farm land received too minimum irrigation water from the sources. This result shows Arba Minch irrigation scheme received an additional 100 % irrigation water compared with the irrigation required as presented in Fig. 10a and b. Based on [55], the excessive irrigation can also lead to the leaching of vital nutrients from the soil that are essential for crop growth and Arba minch irrigation scheme in lower Kulfo catchment was subject to this problem. Untreated wastewater irrigation introduces contaminants, degrading soil and reducing yields [56], thus threatening Arba Minch University and Kolla Shara farmlands with these issues (Fig. 10c and d). To improve irrigation adequacy, prioritize water distribution efficiency, enhance resource management, and ensure equitable water access [57], focusing on areas like Kollashara farmland with low adequacy levels.

**Table 4:** Adequacy of irrigation (PA) in the study area.

Month	Arba Minch irrigation scheme	Arba Minch University farmland	Private farmland	Kollashara farmland
January	1	0.5	0.4	0.1
February	1	0.6	0.5	0.2
March	1	0.4	0.3	0.1
**Mean**	**1**	**0.5**	**0.4**	**0.1**

Based on [58], irrigation adequacy is classified as very good with a performance adequacy (PA) between 0.90 and 1.0, good with a PA from 0.80 to less than 0.90, and poor with a PA below 0.80. The Arba Minch irrigation scheme falls under the very good category, while the remaining users of irrigation in the area of present study were classified under poor performance. The University and private farmlands also receive irrigation, though at lower levels, suggesting some irrigation but possibly not sufficient for optimal crop growth, depending on their crop requirements. Kollashara farmland receives the least irrigation, indicating potential water stress and the maximum irrigation demand was observed in February. The University and private farmlands also see an increase, suggesting improved but potentially still sub-optimal irrigation levels. Kollashara farmland also sees an increase but remains the lowest, indicating continued water stress. March shows a slight decrease in irrigation amounts compared to February but generally remains consistent with February's pattern. The Arba Minch irrigation scheme maintains a relatively high level of irrigation, ensuring continued adequate water supply. However, the University and private farmlands receive less irrigation compared to February, possibly indicating some level of water stress, especially if crop water requirements increase during this period. Kollashara farmland continues to receive the least irrigation, potentially facing significant water stress.

#### 3.6.2 Dependability (PD)

**Fig. 11:**
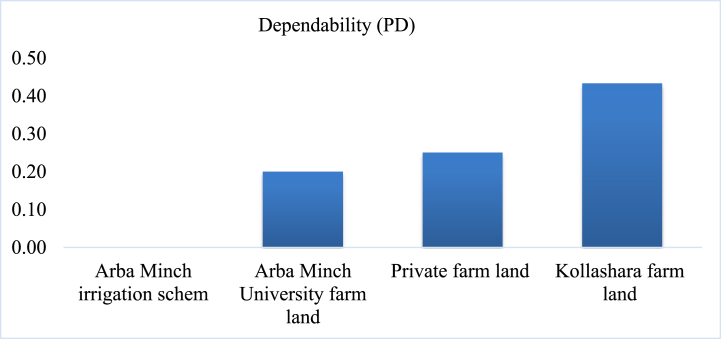
Irrigation dependability (PD) in the study area.

Based on [59]*,* irrigation dependability is classified three ranges: good (0–0.1), indicating optimal conditions, fair (0.11–0.2), indicating acceptable conditions, and poor (>0.2), indicating suboptimal conditions. The Arba Minch irrigation scheme was classified as having good dependability with 0 irrigation dependability. Arba Minch University farmland was classified as fair, and the private farmland and Kollashara farmland were classified as poor dependability (Fig. 11). The mean value of irrigation dependability at the upper, mid, and lower location of the Tahtay Tsalit irrigation scheme in the northern Ethiopia was ranged from 0.03 to 0.08 with an average of 0.057 [60]. The mean value of dependability of the Hare irrigation scheme in the Arba Minch region was 0.05 (head reach), 0.3 (middle reach), and 0.27 (tail reach) [37]. The maximum variation flow adequacy in the current study was found under the upper reach of the scheme but the maximum variation of irrigation adequacy was found under the mid, and lower reach of the hare irrigation scheme.

#### 3.6.3 Equity (PE)

**Fig. 12:**
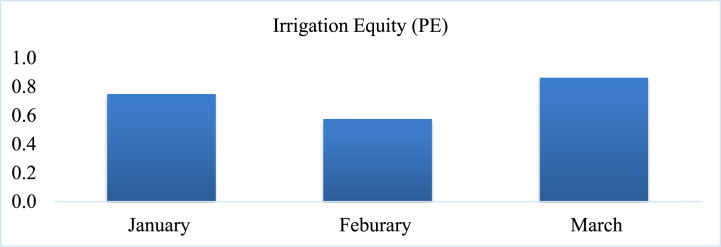
Irrigation equity (PE) in the study area.

The average value of irrigation equity was greater than one especially in February and March which indicates the amount of delivered irrigation water to the field was highly variable in the area of present study during the month of irrigation season (Fig. 12). The good in equity encompasses values ranging from 0 to 0.1, indicating optimal or desirable conditions. Values falling between 0.11 and 0.25 are categorized as fair, suggesting acceptable but moderately less favourable conditions compared to the good category, and values exceeding 0.25 are classified as poor indicating conditions that are considered suboptimal or inadequate [61]*.* Therefore, the implication of the present investigation suggests that the irrigation scheme in the study area was found under poor conditions of irrigation equity with a large value, and the maximum value was observed in March. The average equity of the Hare irrigation scheme in the Arba Minch region was 0.23 (head reach), 0.32 (middle reach), and 0.48 (tail reach) [37] which was lower compared with the value of the current study.

#### 3.6.4 Conveyance efficiencies

**Fig. 13:**
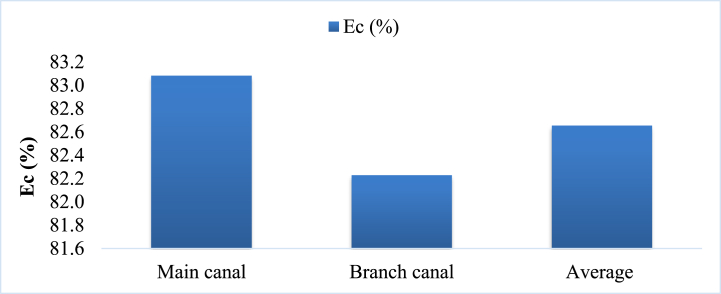
Conveyance efficiencies of irrigation canal (Ec) in the study area.

The conveyance efficiencies of the irrigation canal varied from 82.2 (unlined branch canal) to 83.1 % (unlined main canal) as presented in Fig. 13. Based on [62]*,* conveyance efficiencies for unlined clay compacted irrigation canals vary based on length: for canals longer than 2 km, the recommended efficiency is 80 %; for lengths between 0.2 km and 2 km, it increases to 85 %; and for canals shorter than 0.2 km, the efficiency recommendation rises to 90 %. The conveyance efficiency of the branch canal in the area of present study was minimal compared with the expected value and the main canal also showed good performance status. The average values of conveyance efficiency of unlined main and branch canals of the Lemchek-Sewur irrigation scheme in North Shewa were 86.2 % and 86.3 %, respectively [18] and this scheme has good performance compared with the current study. The mean conveyance efficiencies of the unlined main canal of the Chiro, and Sewur irrigation schemes in Ethiopia are 82.7 % and 77.9 %, respectively [63] which was lower compared with the value present study. The irrigation canal system in lower Kulfo catchment was showed variable efficiencies, with the main canal performing well and the branch canal below benchmarks. This highlights the need for tailored management to optimize water conveyance and ensure sustainable irrigation practices.

**Fig. 14:**
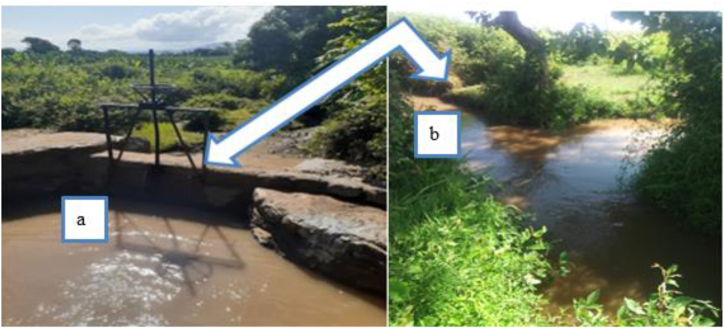
Water loss through canal escape (a) and traditional offtake (b).

The causes irrigation water loss nonfunctional canal escape (Fig. 14a) and traditional water control methods such as mud, stone, and leaves (Fig. 14b). Lining the canals, providing modern water control structure and regular maintenance can enhance conveyance efficiency, improving performance and meeting recommended standards for various canal lengths [64].

#### 3.6.5 On-farm performance efficiencies

**Fig. 15:**
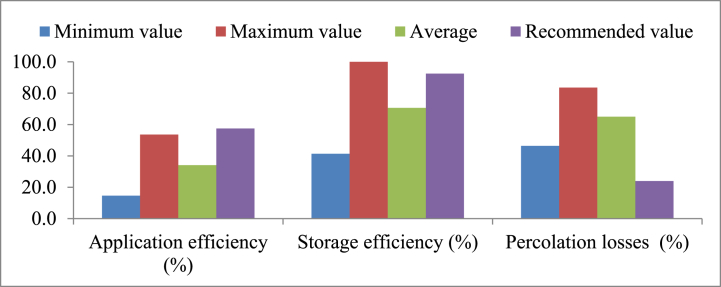
Minimum, maximum, average, and recommended on-farm irrigation efficiencies in the lower Kulfo catchment irrigation scheme.

The mean value application efficiency in the present study was 34.1 % (Fig. 15) which was minimum compared with expected value of 57.5 % [65]. The mean storage efficiency was 70.1 %, which is less than the recommended storage efficiency of 92.5 % [66]. Irrigation was applied by guess without any monitoring of soil moisture depletion and irrigation interval and adapted flooding irrigation in the current study was resulting low irrigation application efficiencies. The mean value of percolation loss of the Gemesha and Ufute irrigation schemes were 41 % and 31 % [67], respectively. Nevertheless, the evaluated value of percolation ratio in the current study was 65 %. This high percolation loss was due poor slope adjustment along the furrow length. This percolation loss problem in the scheme to be minimize by, enhance furrow/border design, use check structures, apply mulch, schedule irrigation precisely, and adopt modern techniques [68].

The average overall irrigation efficiency in the current study was 28.2 %, while the Cheleleka irrigation system in the Rift Valley Lake Basin of Ethiopia had an efficiency of 17 %. This efficiencies of the present study and the Cheleleka irrigation system were found under poor performance status when compared with the expected value of 55 % [44]. This low overall irrigation efficiency was observed due to a lack of scientific-based irrigation water application. According to Ref. [65], irrigation schemes are categorized as good, reasonable, and poor, with anticipated overall irrigation efficiencies of 50–60 %, 40 %, and 20–30 %, respectively and the present falling within the poor category.

### 3.7 Water and land productivity

The mean value of water productivity and yield per irrigable land of onion, wheat, pepper, watermelon, and maize in the present study were 0.94 kg/m^3^ & 4.9ton/ha, 0.07 kg/m^3^ & 0.5ton/ha, 1.5 kg/m^3^ & 6.2ton/ha, 0.5 kg/m^3^ & 0.6ton/ha and 0.9 kg/m^3^ & 4.2ton/ha, respectively. As reported by Ref. [69], the value of total yield per irrigabale land and water productivity of onion under control irrigation practics in the Arba Minch area were 25ton/ha & 3.2 kg/m^3^, respectively and the value of present study was minimum compared with this earlier finding. Based on [70], the land productivity of wheat under full irrigation in the lower Kulfo catchment was 2.7ton/ha, which is higher compared to the productivity achieved with the current study or existing traditional irrigation scheme. The yield of watermelon and maize per unit of land and water under control irrigation were 5.7 kg/m^3^ & 12.8ton/ha [47], and 1.05 kg/m^3^ & 7.8ton/ha [71], respectively. Control irrigation with water level application can enhance water and land productivity by 11.4 times, and 21.3 times, respectively, compared with value of current study. Similarly, land and water productivity of maize in the current study were reduced by 3.6ton/ha& 0.15 kg/m^3^, respectively compared with earlier control irrigation practices. This indicates that traditional irrigation methods have a significantly negative impact on crop productivity and implementing control irrigation in the area of current study can improve productivity of irrigation scheme [72].

## 4 Conclusion and recommendation

The irrigation practices in the Lower Kulfo catchment do not align with management's allowable depletion, resulting in excess water loss in the field. This mismanagement causes soil water storage levels to exceed field capacity, leading to waterlogging. The current irrigation practices in the Lower Kulfo catchment adopted shorter irrigation intervals leads to frequency of irrigation, which can exacerbate issues such as surface water pondage. The Arba Minch irrigation scheme consistently receives 80–100 % extra irrigation water each month, demonstrating perfect adequacy and low variability in irrigation adequacy. In contrast, remining user of irrigation in the area of Lower Kulfo were faced to water scarcity problems, with high variability in both temporal and spatial irrigation adequacy. Kolla Shara farmland was more affected compared with other middle and tail reach of the scheme, receiving only 10 % of its required irrigation water. On-farm irrigation efficiencies, including application and storage, are significantly below recommended values, leading to insufficient crop productivity compared with control irrigation practices in the region. To improve irrigation practices in the Lower Kulfo catchment, adopt a 7-day irrigation interval along with application of optimum amount based on the management allowable depletion for each crop, reducing waterlogging and improving soil moisture levels. Ensure equitable water distribution to address water scarcity, particularly in areas like Kolla Shara. Invest in infrastructure and training to enhance irrigation efficiencies, thereby boosting crop productivity to meet regional standards [73].

## 5 Data availability statement

Data will be made available on request.

## Additional information

For this paper, there is no available further information.

## CRediT authorship contribution statement

**Birara Gebeyhu:** Reta, Formal analysis, Data curation, Conceptualization. **Samuel Dagalo:** Hatiye, Writing – original draft, Data curation. **Mekuanent Muluneh:** Finsa, Software, Formal analysis.

## Declaration of competing interest

The authors state that they do not have any known competing financial interests or personal ties that could appear to have influenced the work disclosed in this study.
